# A Hand-Book of Pathological Anatomy, and Histology, with an Introductory Section on Post-Mortem Examinations, and the Methods of Preserving and Examining Diseased Tissues

**Published:** 1893-03

**Authors:** 


A HAND-BOOK OF PATHOLOGICAL ANATOMY, AND HISTOLOGY, with an Introductory Section on Post-Mortem Examinations, and the Methods of Preserving and Examining Diseased Tissues. By FRANCIS DELAFIELD, M. D., LL. D., Professor of the Practice of Medicine, College of Physicians and Surgeons, Columbia College, New York, and T. Mitchell Prudden, M. D., Professor of Pathology and Director of the Laboratories of Histology, Pathology, and Bacteriology, College of Physicians and Surgeons, New York. Fourth edition, illustrated by 300 wood engravings, printed in black and colors. Pp. 715. New York : William Wood & Company. 1892.

The fourth edition of this excellent work needs but little introduction to the profession, inasmuch as the former editions have enjoyed a wide circle of professional readers. This edition is some 100 pages larger than the third edition, many chapters having been rewritten and enlarged upon. Many drawings and photographs have been added, some showing the presence of microörganisms in the diseased tissues, adding greatly to the value and interest of the book.

The third edition was reviewed at some length in the March (1890) number of the JOURNAL. The typographical work of this is even better than in the former edition, thanks to the enterprise of its publishers. W. C. K.

